# Benchmarking selected computational gene network growing tools in context of virus-host interactions

**DOI:** 10.1038/s41598-017-06020-6

**Published:** 2017-07-19

**Authors:** Biruhalem Taye, Candida Vaz, Vivek Tanavde, Vladimir A. Kuznetsov, Frank Eisenhaber, Richard J. Sugrue, Sebastian Maurer-Stroh

**Affiliations:** 1 Bioinformatics Institute, A*STAR, 30 Biopolis Street #07-01 Matrix, Singapore, 138671 Singapore; 2 School of Biological Sciences, Nanyang Technological University, 60 Nanyang Drive, Singapore, 637551 Singapore; 3 Aklilu Lemma Institute of Pathobiology, Addis Ababa University, P.O.BOX 1176, Addis Ababa, Ethiopia; 4 Department of Biological Sciences, National University of Singapore, 8 Medical Drive, Singapore, 117597 Singapore; 5 School of Computer Engineering, Nanyang Technological University, 50 Nanyang Drive, Singapore, 637553 Singapore; 6 National Public Health Laboratory, Ministry of Health, 3 Biopolis Drive, Synapse #05-14/16, Singapore, 138623 Singapore; 7 Institute of Medical Biology, A*STAR, 8A Biomedical Grove, #06-06 Immunos, Singapore, 138648 Singapore

## Abstract

Several available online tools provide network growing functions where an algorithm utilizing different data sources suggests additional genes/proteins that should connect an input gene set into functionally meaningful networks. Using the well-studied system of influenza host interactions, we compare the network growing function of two free tools GeneMANIA and STRING and the commercial IPA for their performance of recovering known influenza A virus host factors previously identified from siRNA screens. The result showed that given small (~30 genes) or medium (~150 genes) input sets all three network growing tools detect significantly more known host factors than random human genes with STRING overall performing strongest. Extending the networks with all the three tools significantly improved the detection of GO biological processes of known host factors compared to not growing networks. Interestingly, the rate of identification of true host factors using computational network growing is equal or better to doing another experimental siRNA screening study which could also be true and applied to other biological pathways/processes.

## Introduction

Gene and/or protein networks are used as a cardinal representation of various types of biological processes and help in the prediction of molecular and cellular function^1^. It has been indicated that interacting genes/proteins may be part of the same pathway or biological process and, in larger scale, work together in similar cellular functions^2, 3^. Reliable prediction and precise treatment of complex cellular functions require knowing the network of as many as possible genes and their products (e.g., proteins) connected in functional pathways. Pairwise interactions at the gene and protein levels have been analyzed extensively, often from low or high-throughput experimental screens, with the links (annotated as functional interactions) available in online databases (reviewed in ref. 4). However, these methods do not fully investigate the highly complex interaction patterns in cellular systems and variability in terms of their accuracy and reproducibility^5^ is encountered. Additionally, interactions seen *in vitro*, especially in large-scale screens, may not occur *in vivo* due to spatial and temporal constraints. At the same time, the exact number of protein-protein interaction (PPI) in human is not known, and the available data is estimated to represent approximately 10% of the total PPIs in human^6^. Hence, computational PPI predictions have become increasingly important for detection of new interactions and protein networks. There are several computational gene and/or protein network databases that combine different experimental and computationally predicted interactions through the integration of PPI information that are obtained from different sources (reviewed in refs 7–9).

Network growing functions allow extension of networks with additional related genes and are important to improve the understanding of the greater functional context and organization of genes and/or proteins, as well as for discovering novel functions of genes that could have been missed by experimental investigations^10^. This could also be achieved by integrating gene expression or “omics” data from specifically investigated conditions with PPI information into a complete large network followed by extraction of active sub-networks related to the respective conditions^11^. The non-expressed genes which could be identified in the network are expected to have similar function with the expressed genes that could be missed by the experiments^11^. Such sub-network extraction tools, given user input of gene expression data, include DEGAS (DysrEgulated Gene set Analysis via Subnetworks)^12^, KeyPathwayMiner^11^, and JActiveModules^13^. While other methods (e.g. Search Tool for the Retrieval of Interacting Genes/Proteins (STRING)^8^, GeneMANIA^9^ and Ingenuity Pathway Analysis (IPA) (http://www.ingenuity.com/products/ipa)^14^ support the construction of networks for query gene lists without explicit user-provided expression data and automatic network growing of specific numbers of additional genes for discovering functionally related genes^15–17^.

The motivation of this study was to benchmark the selected gene/protein network growing services STRING^8^, GeneMANIA^9^ (both academically freely available) and IPA^14^ (commercialized) in the context of virus-host interaction (e.g. Influenza A virus (IAV)) in a user-relevant setup. These three services were selected for their ability to take user gene lists as sufficient input and execute the network growing with user-defined numbers of nodes automatically added to the query genes (e.g 10, 20, 50 and 100 nodes).

It would be theoretically interesting to compare the tools’ basic algorithms in a well-defined equal search space. However, it would not reflect the reality that a characteristic difference of the tools is the use of different underlying interaction sources and information, which will directly influence the performance a typical user will experience. With the benefit of the user community and intrinsic inseparability of algorithms and used databases in mind, our aim was to benchmark the available tools in their full implementation as tool/web service.

We chose influenza virus required host factors (IHFs) interactions for this benchmark because (1) several large-scale screens have been performed recently with data available, (2) experimentally validated host factors do not correspond to a single pathway but are functionally loosely linked covering a broad range of cellular functions and (3) network growing services can be used to find new candidate host factors as potential drug targets against influenza in future.

## Results

### Network growing algorithms partially recover IHFs from siRNA screens

Prior to embarking on the network growing benchmark, we performed a complete re-analysis on known IHFs from several siRNA screening studies. Overall, 1,580 human IHFs were identified from 11 siRNA studies of which 28 and 158 IHFs were shared by at least three and two studies respectively (Supplementary Fig. S1a,b and Supplementary Data 1). Mapping of these IHFs to all three network growing tools for fair comparison was successful for 28 of the 28, 153 of the 158 and 1463 of the 1,580 IHFs (Supplementary Fig. S1a). Next, we used the 28 (small set) and 153 (medium set) IHFs as query genes (seed sets) to grow with 10, 20, 50 and 100 additional genes in each of the network growing tools. The intersection of grown genes from small set seeds were evaluated against the 153 and 1463 IHFs, while the grown genes from medium set seeds were evaluated against the whole positive sets (1463) IHFs.

**Figure 1:**
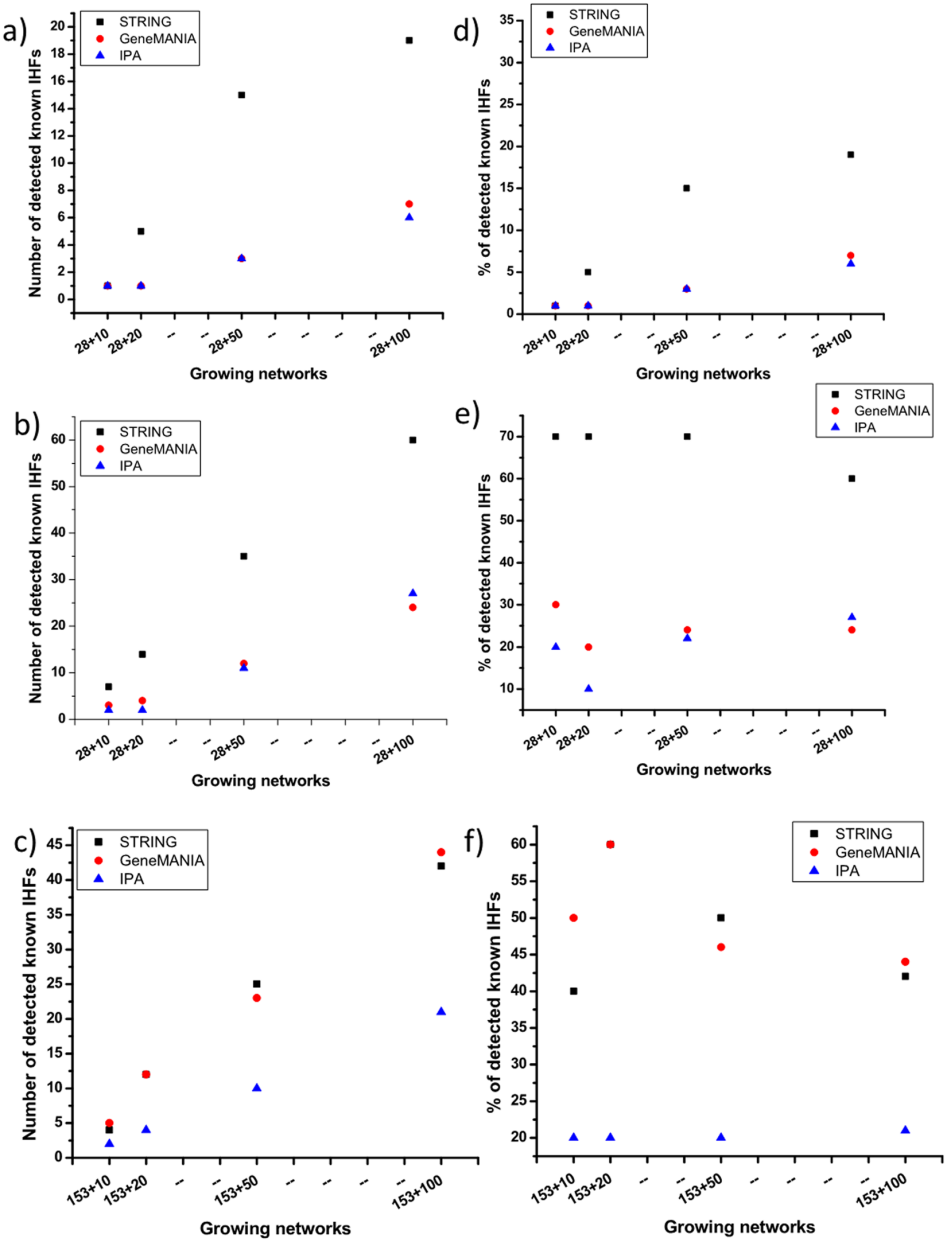
The trend of network growing tools in detecting known IHFs. (**a**) Number of detected known IHFs upon growing networks with the small set (28 IHF) seeds and 153 known IHFs as positive set. (**b**) Number of detected known IHFs upon growing networks with the small set (28 IHF) seeds and 1463 known IHFs as positive set. (**c**) Number of detected known IHFs upon growing networks with medium set (153 IHFs) seeds and 1463 known IHFs as positive set. (**d**) Percentage of detected known IHFs upon growing networks with the small set (28 IHF) seeds and 153 known IHFs as positive set. (**e**) Percentage of detected known IHFs upon growing networks with the small set (28 IHF) seeds and 1463 known IHFs as positive set. (**f**) Percentage of detected known IHFs upon growing networks with medium set (153 IHFs) seeds and 1463 known IHFs as positive set.

The results showed that as the number of genes building the network increases from 10 to 100, the number of genes intersecting with known IHFs also increased (Fig. 1a,b and c). However, the rate of IHFs detections (percentage) shows variable trends with slightly decreasing performance after growing 20 and/or 50 (Fig. 1d,e and f), suggesting early recruitment of known host factors. Among 100 genes grown from the small set seeds, 60%, 24% and 27% of the automatically recruited genes in STRING, GeneMANIA and IPA respectively, overlapped with the known IHFs (Fig. 1e). Similarly, the detection rates were 42%, 44% and 21% in STRING, GeneMANIA and IPA, respectively with the medium set seeds (Fig. 1f). Therefore, while using the small set as seed genes, STRING was the sole strongest performer (Fig. 1e), both GeneMANIA and STRING seem to perform equally well with the medium set seed genes (Fig. 1f).

Among the 100 genes recruited by growing networks using the 28 small set seeds, we also examined the distribution of correctly identified IHFs among the 125 genes with support by 2 siRNA studies vs 1,310 genes with support by only one siRNA screen (Supplementary Fig. S1a and Fig. 1a and d). For a gene to be detected in the 125 shared lists, proportionally we expect ~10 genes to be detected in the 1,310 non-shared genes. Interestingly, the proportions of shared genes detected in STRING, GeneMANIA and IPA were 3.4, 3.1 and 2.4 times higher than the non-shared genes respectively. This means that the network growing tools recruit shared IHFs in higher proportions, suggesting the reliability of the methods for identification of highly relevant IHFs candidates.

### The network growing tools recruit other functionally related IHFs

**Figure 2:**
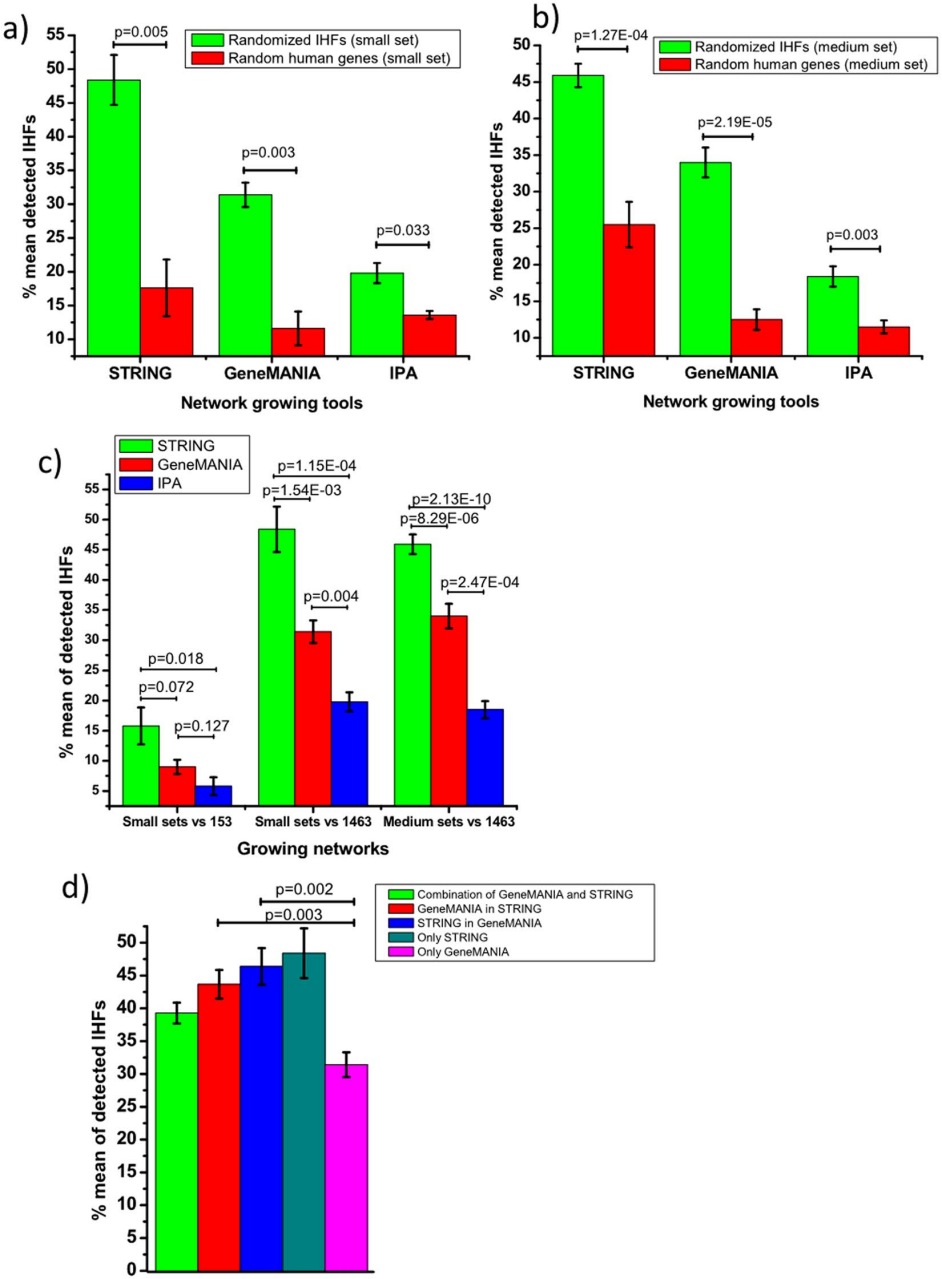
Detection rate of network growing tools after randomization of IHFs and random human proteins. (**a**) Comparison of the detection performance of the network growing tools after growing 100 genes using either small set (30 genes) IHFs or random human proteins as seeds and 1463 IHFs as positive sets. P-values are the result of a paired Student-t test analysis. (**b**) Comparison of the detection performance of the network growing tools after growing 100 genes using either medium set (146 genes) IHFs or random human proteins as seeds and 1463 IHFs as positive sets. P-values are the result of a paired Student-t test analysis. (**c**) Detection performance comparison of the three network growing tools (**d**) The detection rate of STRING and GeneMANIA after combination and interchange of the 1^st^ 50 grown genes.

To verify that the performance of the network growing tools is neither by chance nor biased by generally highly connected nodes in human interaction networks, we randomized the IHFs into two groups, (1) the 153 IHFs were randomly distributed into 5 non-redundant groups with ~30 genes each (small set IHFs), and (2) the 1463 IHFs were randomized into 10 non-redundant groups each containing ~146 genes (medium sets) (Supplementary Data 1). Similarly, we randomly selected equal numbers of small set and medium set genes from the whole pool of human genes (19,004 genes) obtained from HUGO Gene nomenclature committee (HGNC) database^18^ (Supplementary Data 1). The result showed that in both small (Fig. 2a) and medium (Fig. 2b) set genes the percentage mean detection of known IHFs was significantly higher with IHF seeds compared to the seeds from random human genes (Fig. 2a and b). This suggested that given IHFs as seeds, the detection of other IHFs by using these network growing tools was not likely to be by chance but may indeed reflect relevant functional connections.

### Performance of IHFs detection varies between the network growing tools due to different data sources used

Using the randomized subsets, we could also benchmark statistical significance of the different detection rates of IHFs by the network growing tools confirming highest performance by STRING followed by GeneMANIA and IPA (Fig. 2c). The advantage of STRING was most dominant for both the smaller and medium sets, while GeneMANIA had relatively small improvement of performance when the medium set was used (Figs 1b,c and 2c).

To gain more insight on the differences in the performance, we investigated the grown gene differences, and the effect of edges (data sources) and network topological parameters. Pairwise analysis of the grown genes from the small and medium set IHFs seeds from each network growing tool showed that among the 1966 grown genes (from all network growing tools (Supplementary Data 1)), only 3.7% were shared by all three tools and high proportions of distinct genes were being grown by IPA (40.3%) followed by GeneMANIA (29.6%) and STRING (7.9%) (Supplementary Fig. S2a). These differences could be due to several reasons, including data sources as well as the growing algorithms used by the three tools. It was difficult to directly compare the tools over the same search space restricted to the same underlying database because their success and performance perceived by the user (the aim of our benchmark) depends critically on the combination of their unique and different sets of edge sources. Nevertheless, we tried to evaluate the performance of the three tools in a setup of closest possible search space where the original source databases are largely overlapping, which is the case for protein-protein interaction data covered by edge info labels “experiment and database” for STRING, “physical interaction” for GeneMANIA and “experiment” for IPA (Supplementary Data 1 and Supplementary Fig. S2b). However, also only using this overlapping subset of the source data, there continues to be a difference in performance of the tools. One factor contributing to this is that even in cases where the same original source database was used, e.g. BioGRID, there can be different interpretation and hence consideration of edge data in the different tools. However, STRING and GeneMANIA performed similarly when co-expression data is used as the only data source (Supplementary Fig. S2b). This suggested that the experimental protein-protein interaction database in STRING may play a big role in its performance.

To study this further, we compared the contribution of each annotated edge type on the respective tools’ performance. This analysis was limited to STRING and GeneMANIA as IPA didn’t provide results by edge type as direct output. The result showed that analyzing with only “experimental” edge was almost as powerful as the performance of STRING default considering all edge types (Supplementary Fig. S2c). Similarly, using only co-expression had similar performance as the complete GeneMANIA suite, with additional strong individual performances of edge types “Predicted” (protein-protein interaction including annotation transfer from orthologues in other organisms) and “Pathway” (Supplementary Fig. S2d).

Comparison of the mean scores of grown genes intersecting or non-intersecting with known IHFs somewhat reflects comparing known true positives with unknown true plus false positives. Interestingly, GeneMANIA was significantly better than STRING in separating these two sets when intersecting with the smaller set of 153 known IHFs that are shared at least by two studies, while it is the other way round in favour of STRING when intersecting with the complete 1463 known set. This occurs because the genes non-intersecting with the small known set, but predicted by STRING, are in fact good candidates intersecting with the bigger known set (Supplementary Fig. S2e,f).

### Network topology analysis shows characteristic differences between networks from different tools

Network topology analysis indicated that the mean values of several network topological parameters were significantly different between STRING and GeneMANIA. The mean values of average shortest path length, clustering coefficient, eccentricity, radiality and topological coefficient were significantly higher in STRING when compared with GeneMANIA (Supplementary Data 1). In contrast, closeness centrality and degree averages were higher in GeneMANIA when compared with STRING (Supplementary Data and Supplementary Fig. S3a). Upon investigation of the degree distribution, the higher degree average in GeneMANIA originates from a long tail made up of several very high degree nodes (Supplementary Fig. S3b). It was interesting to note higher degrees in GeneMANIA but higher clustering coefficient in STRING. This was, because the degree of a node represents simply the number of immediate neighbours it has, while the clustering coefficient measures the same plus further interconnectivity of these immediate neighbours with each other. The latter could be high when the edge data comprises physical interaction clusters typical for protein complexes which may be a dominant edge source for STRING. We also computed the topological properties of the intersecting and non-intersecting grown genes, and the result showed that the mean score of betweenness centrality and stress in intersecting grown genes (known IHFs) were consistently higher compared to the non-intersecting grown genes (Supplementary Data 1). Nodes with higher stress and betweenness centrality values have a critical role in overall network connectivity and often connect clusters or cellular processes. Future detailed follow-up work will have to establish if this can be exploited to improve identification of known host factors or it simply reflects that critical hubs are more likely to be found in phenotypic screens which are the source of our known set.

### Interchanging and combination of genes didn’t improve the performance of the free tools

As many grown genes by the network growing tools are distinct, we tested if combination and interchange of the first 50 genes from GeneMANIA and STRING (free web services/tools) could improve the detection rate of known IHFs. First, we tried combining the first 50 genes of all pairs of the methods to get a total of 100 genes. However, the performance of the combination of genes from the two network growing tools doesn’t exceed the performance of 100 genes from STRING alone (Fig. 2d). Next, we tried interchanging gene sets by taking the first 50 genes from one method as a seed along with the randomized 30 IHFs to a second method for growing by another 50 genes to complete 100 grown genes (see materials and methods for detail). The result showed that GeneMANIA with the first 50 from STRING as input seems better compared to GeneMANIA alone (Fig. 2d). However, it was again lower than the detection rate of STRING alone, and it appeared that the number of genes contributed by STRING correlated with the strength of performance.

### Network growing algorithms detect GO biological process and pathways of the known IHFs

**Figure 3:**
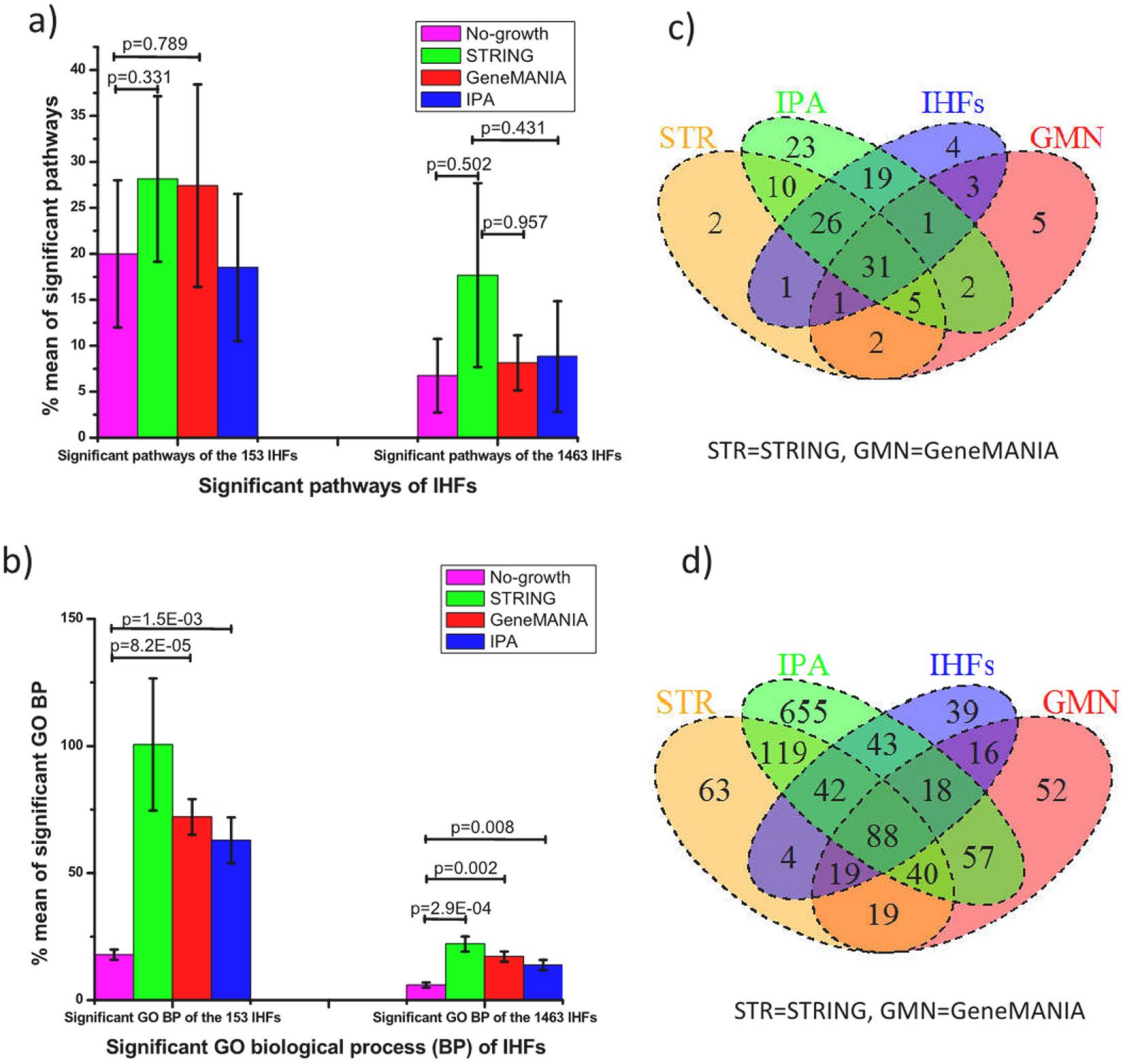
Performance comparison of the three network growing tools in terms of KEGG pathways and GO BP. (**a**) Rate of overlapping KEGG pathways before and after network growing relative to pathways of the 153 and 1463 known IHFs. (**b**) Rate of overlapping GO BP of the grown genes relative to GO BP of the 153 and 1463 known IHFs. (**c**) Pairwise analysis of KEGG pathways from the grown genes in the network growing tools and KEGG pathways of the positive sets (1463 IHFs) (**d**) Pairwise analysis of GO BP from the grown genes in the network growing tools and GO BP of the positive sets (1463 IHFs).

As described above, the network growing tools have the potential to detect IHFs known from siRNA screens at rates as high as 60%. We extended the analysis into benchmarking the performance of the network growing tools at the GO biological process (BP) and Kyoto Encyclopedia of Genes and Genomes (KEGG) pathway analysis level. Database for Annotation, Visualization and Integrated Discovery (DAVID) v6.8^19^ was used for analysis using the seed genes before and after growing 100 genes in the respective network growing tools. As the true positive list of pathways, we defined all those found when using either 153 or the combined set 1463 IHFs as input. We found that the mean percentage of correctly identified pathways after growing the networks was higher in STRING (both from 153 and 1463 IHFs pathways) and GeneMANIA (for 153 IHF pathways) than before growing networks, but not statistically significant (Fig. 3a). It should also be noted that more than 70% pathways from the grown 100 genes in GeneMANIA and STRING were true positives (Supplementary Fig. S3c), which was considerably better than the recall rates of genes (31.4 to 48.4%, Fig. 2c). In contrast, the intersection of GO BP after growing a network was significantly higher compared to before growing the networks (Fig. 3b). This is not too surprising, since the tools may include edge sources correlating with GO similarity in the growing process. Similar to the KEGG pathways more than 60% of the GO BP of the grown genes in GeneMANIA and STRING were intersecting with true positive set pathways (Supplementary Fig. S3d).

When comparing the overlap of KEGG pathways (Fig. 3c) and GO BP (Fig. 3d) identified after network growing, we observed several shared but also some unique pathways or BPs suggested by the different tools. Matching the underlying biology, several of the intersecting pathways were implicated in supporting different stages of the IAV replication cycle^20–22^. In addition, the three network growing together identified 5 and 40 novel pathways and GO BP respectively (Fig. 3c and d and Supplementary Data 2) that could be investigated further.

### Network growing approach on existing data could be as powerful as new siRNA screens for finding relevant additional IHFs

**Figure 4:**
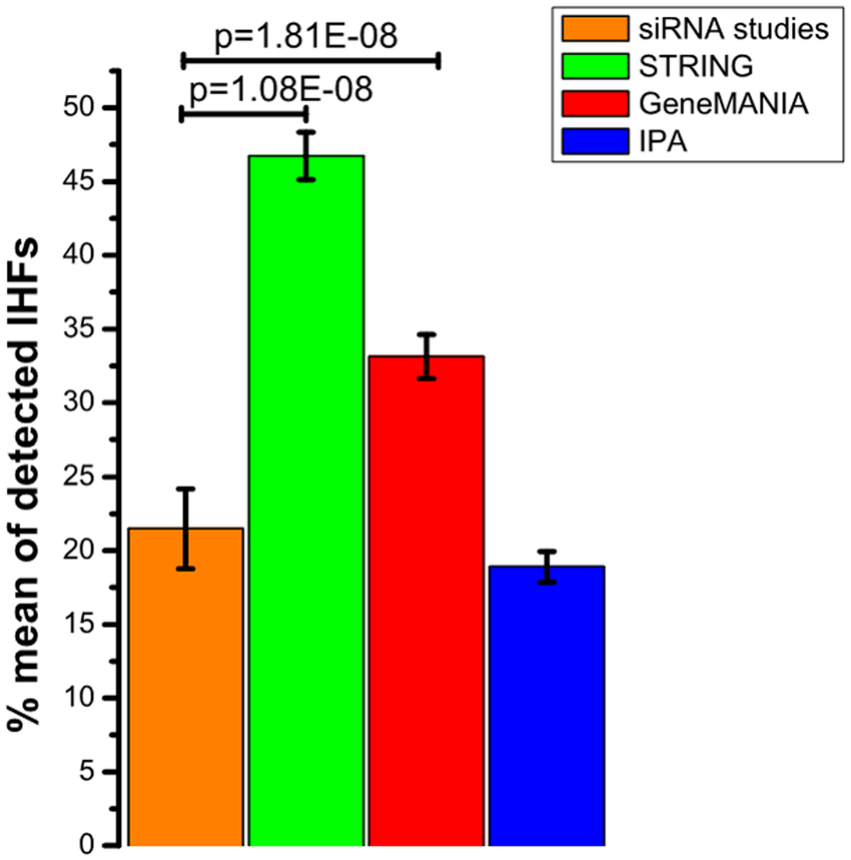
Comparison of siRNA experimental studies and network growing tools in detecting known IHFs.

The overlap of IHFs from a single siRNA screen with any of the other siRNA screens ranged between 11. 1% and 43.1%, with a mean percentage of 21.5% (Supplementary Data 1). However, the mean overlap of the IHFs retrieved from growing networks with any other siRNA screen was 46.7, 33.1 and 18.9 for STRING, GeneMANIA and IPA (Fig. 4) respectively. This leads to the interesting conclusion that computational network growing approaches on existing data could be as powerful as new experimental siRNA screens in finding additional relevant IHFs.

## Discussion

In this study, we compared the performance of selected computational gene network growing tools with each other using a large set of 1,463 experimentally determined influenza host factors. This set is well suited since the siRNA screen data has not yet directly been exploited by the tools, and the genes were found to cover a broad range of representative cellular functions. Considering the experimentally determined genes as true positives, the detection percentages are equivalent to a measure of precision or positive predictive value of the methods.

We need to emphasize that network growing tool performance is intrinsically linked to the respective underlying data types and data sources used, which is also apparent in our benchmark results. Besides the differences in underlying databases between the tools, we have also observed considerable performance fluctuations of different versions of the same tool after database updates which highlight the clear dependency of this approach on the available search space (e.g., Fig. S3e).

Furthermore, another main difference between the tools was that they not only use protein-protein interactions, but they also use other interaction/edge types such as co-expression, gene neighbourhood, co-localization, shared domains, text-mining, genetic interaction etc which are mostly unique to the different tools. Since the contribution of the different edge type sources may be of interest to understand why certain tools perform better, we provided a detailed comparison of the individual edge type contributions to prediction performance (Supplementary Data 1 and Fig. S2b,c,d) and found that protein-protein interaction data in STRING seems to play a dominant role in overall performance while for GeneMANIA co-expression is the main contributor. Interestingly, using co-expression alone performed equally well when used through STRING or GeneMANIA which suggested that the basic algorithms used to recruit genes may perform similar if the same underlying data type and set would be used. However, each tool also uses different interpretations for reliability and rules applied to define edges even from the same dataset. Network topology analysis further revealed characteristic differences of the networks produced by the two tools in parameters such as average degree, closeness centrality and cluster coefficient mostly reflecting the differences in approach and underlying source data. Interestingly, some network parameters seem to distinguish between true hits and assumed false positives, which will require further testing, but could inspire future method development.

There may be concerns that the network growing approach could select highly connected genes in general and not necessarily those specifically related to the input set. To confirm that this is not the case, we compared the network growing performance of the influenza-related host seed genes with that of random human genes and find a significant difference in favour of growing influenza-related rather than the highly connected but random genes.

Although there are considerable numbers of distinct genes suggested uniquely by some methods but not others, the interchange and combination of genes recruited from GeneMANIA and STRING did not improve the performance of host factor detection rates. Although the performance was not improved, interpretation of results from a single method may lead to biases in interpretations and considering multiple methods could uncover a more diverse and complete set of host factors albeit at the same time diluted with false positives.

Overall, the network growing tools had better recall rates at GO Biological Process and KEGG pathway level than for genes. This was expected, since several of the tools directly use this information for growing networks and it was also reported previously that influenza host factors (IHFs) identified with siRNA screens in different studies have higher overlap at functional categories than at gene level^23^.

The main goal of the previous influenza siRNA screens was to identify druggable host targets. There is currently a limited availability of drugs to treat and prevent influenza virus infection, which is compounded by high mutation rates that lead to drug resistance. Drug repurposing is a recent strategy that has been proposed as one possible solution to this problem. In this strategy, existing drugs that are in clinical use and that have known safety profiles are evaluated for either anti-viral activity or as therapeutics to treat virus infection. System-wide phenotypic data together with bioinformatics methods have been used for *in silico* prediction for repurposing FDA approved drugs as alternative therapeutics against infectious diseases and cancer^24, 25^. Therefore, it would be plausible that network growing could identify new host factors that could be suggested as anti-influenza targets. Indeed, applying this approach on newly identified candidate host targets from network growing (Fig. S4a,b and Supplementary Data 3) suggested 258 new predictions for existing FDA-approved drugs for further investigations as potential anti-IAV therapy (Fig. S4c and Supplementary Data 3). Importantly, six drugs that have previously been shown experimentally to have an effect on influenza virus were also identified by our investigation, thereby adding support to the validity of the approach (Fig. S4c and Supplementary Data 3)^20^.

## Conclusion

Our aim was to establish the performance of selected network growing tools in a typical real usage scenario to recover known influenza host factors from siRNA screens not included as source for the tools. All 3 tools tested are able to do so significantly better than expected by chance and even to a similar extent as conducting a new experimental siRNA screen. A detailed comparison of network topology and performance contribution of different data types used by individual tools highlighted that the network growing algorithms crucially depend on their underlying databases and data types that are used. While we used the system of influenza host interactions here, we believe that these network growing tools would be similarly able to recover relevant gene/protein connections also for other biological systems, viruses and diseases.

## Materials and Methods

### Data collection

Influenza A virus host factors (genes) with the same phenotype i.e. either supporting or suppressing IAV replication were collected from siRNA screen studies. A total of 11 published siRNA screen studies were identified which either investigated larger sets consisting of groups of genes (e.g. kinases)^26, 27^ or the whole genome-wide level^20–22, 28–33^ (Supplementary Data 1). There are only limited gene overlaps between the siRNA screening studies and this discordance is mainly due to false negative results^23, 34^. Therefore, a merged list of all the siRNA screening studies in the context of IAV would give the most comprehensive positive set of IHFs. For the studies that used non-human host cells (e.g. DL1^29^ and MDCK cells)^32^ in their experimental design, the human homologues of the corresponding genes were used in the analysis. We note that a single gene may have multiple Entrez IDs and names^19^ and for the sake of consistency we used the “Official gene symbol” for representation of all genes.

### Network growing tools

Three network growing tools STRING^8^, GeneMANIA^9^ and IPA^14^ were used for network growing analysis. STRING uses experimentally well-described genes, proteins and their interactions and a number of computationally predicted interactions such as gene neighborhood (e.g. prokaryotic operons), paired fusion proteins, gene links via common evolutionary histories (phylogenetic profiles), co-transcription regulators, co-expression patterns, text-mining associations, links inferred from transferring interactions of orthologous proteins interacting in another organism and similarity of the protein structures. STRING (version 10) covers more than 2000 organisms, >900 million interactions of 9.6 million proteins and is updated regularly^8^. STRING uses a naive Bayesian algorithm for computing combined scores from different edge types including a correction for the probability of random observation of an interaction adding related genes to grow the query network is based on the closest combined scores^35^.

GeneMANIA uses association data, including protein and genetic interactions, pathways, co-expression and co-localization similarity information and protein domain similarity data. This tool supports the network analysis for nine organisms (*A*.*thaliana*, *C*. *elegans*, *D*. *rerio*, *D*. *melanogaster*, *E*. *coli*, *H*. *sapiens*, *M*. *musculus*, *R*. *norvegicus* and *S*. *cerevisiae*) and uses aggregated interaction links from hundreds of data sets (experimentally validated and/or computationally predicted)^9^. GeneMANIA utilizes two algorithms: (1) a linear regression algorithm to calculate composite functional association network (based on Gene-Ontology (GO) biological process, molecular function and cellular compartment) from multiple networks obtained from different data sources and (2) a Gaussian field label propagation algorithm for predicting gene function from the composite network. For a longer list of query genes (>5) the weights are chosen automatically using linear regression maximizing interaction between seed genes while minimizing interactions of seed genes to other genes not on the query list^9, 36^.

The main attribute of the IPA knowledge database is the high-quality manual curation of texts from peer-reviewed journals and both public and private biomedical databases. The retrieved knowledge is structured into ontologies and the ontology made available as knowledgebase for various applications including functional network analysis. Given user seed genes and the knowledge-base of interaction of thousands of genes with each other, IPA uses a multi-stage, heuristic six step algorithm to produce networks. Briefly, IPA sorts seeds genes based on their interconnectivity to construct multiple small networks. The small networks are then merged by growing with genes from the knowledge-base that can connect the small into bigger networks. Then a p-score is calculated as the probability of finding *f* more seed genes in a set of *n* genes randomly selected from the knowledge-base^37^.

### Growing networks and network analysis

Before growing networks, we first mapped the IHFs to the network growing tools. The IHFs that were mapped to the three tools were used for growing networks. For the network analysis, we first defined two seed sets with different size (number of genes); (1) 28 genes that were shared in more than 2 studies, (2) 153 genes shared by at least two studies (Fig. S1a). Using these two sets of genes as a seed we automatically grew 10, 20, 50 and 100 genes with each of the network growing tools. These numbers (10, 20, 50 and 100 genes) were selected because GeneMANIA uses these fixed numbers for growing networks and the other tools allowed defining any numbers.

The second step of the network analysis was utilizing randomized gene sets as seed base to confirm whether the detection rate was by chance or not. The 153 genes that were shared by two studies were randomized into five groups of 30 genes (small sets (30 A to E)), and the 1,463 genes into 10 groups of 146 genes (medium sets (146 A to J)) (Supplementary Data 1). Each set (either 30 or 146) was used as seed to grow 100 genes in each of the networking tools. In parallel, we randomly selected an equal number of small and medium set genes from 19,006 human genes obtained from HGNC^18^. The grown genes were separated from the seed genes and the newly grown genes from each network growing tool were compared against the list of known IHFs.

The third step of analysis was combining or interchanging grown genes between the network growing tools (STRING and GeneMANIA: as these tools are freely available online) to test if this improves the detection rate of known IHFs. For combination, the first 50 genes from two network growing tools were combined and compared against the list of known IHFs. For interchange, smaller sets of genes were used, and the first 50 grown genes in one tool was used along the seeds to grow additional 50 genes in the other tool. Like the previous analysis, the grown genes from each network analysis were separated from the corresponding seeds and compared against the list of known IHFs.

Topological parameters (average shortest path length, betweenness centrality, closeness centrality, clustering coefficient, degree distribution, eccentricity, neighborhood connectivity, radiality and stress) of the networks from STRING and GeneMANIA were analyzed using Network Analyzer in Cytoscape v 3.4.0^38^.

### KEGG pathway and GO biological process analysis (BP)

KEGG pathway and GO BP enrichment analysis was performed using DAVID version 6.8^19^. First, we did KEGG pathway analysis for small and medium seed sets, as well as the full true positive sets (for 153 and 1463 IHFs). Then, the pathway and GO BP analysis is repeated after growing 100 genes with each respective tool. Significantly enriched (p < 0.05) pathways and GO BP were compared against significant pathways and GO BPs from the true positive sets. In addition, pathway and GO BP for the 100 grown genes by each of the network growing tools were also separately compared with the significant pathways and GO BPs from the positive sets.

### Drug-interaction analysis

Drugs that interact with the known IHFs (1,445 genes) and the new candidate IHFs (1,538 genes) (excluding anti-viral genes) from the network growing analysis were identified using MetaCore^39^. Therapeutic drug-target interactions and secondary drug interactions were obtained via drug-interaction analysis and those drugs with effect on the known or new host targets were extracted if they were FDA approved and only have inhibitory effects on the IHFs. Previously *in silico* predicted or experimentally tested anti-IAV drugs or small molecules were also manually curated from previous reviews and reports (Supplementary Data 3)^20, 40–43^, and were compared using their DrugBank IDs with the current predictions.

### Statistical analysis

Customized Perl scripts were used to refine and count gene lists, for randomization, overlap (pairwise) analysis, and filtering the grown genes from the input seed genes. Percentage of detection for the network growing analysis was calculated as overlap of grown genes with the positive sets of genes (either 153 or 1463 IHFs):

1 $$Percentage\,(Genus)\,( \% )=\frac{Number\,of\,grown\,genes\,in\,each\,networking\,tool\,overlapping\,with\,the\,known\,IHFs}{(Genes)Total\,number\,of\,grown\,genes\,in\,growing\,networks\,(usually\,100)}$$

2 $$Percentage\,( \% )=\frac{Number\,of\,pathways/GO\,BP\,before\,or\,after\,growing\,networks\,overlapping\,with\,positive\,set\,pathways/GO\,BP}{Total\,number\,of\,pathways/GO\,BP\,from\,the\,whole\,host\,factors}$$

3 $$Percentage\,( \% )=\frac{Number\,of\,pathways/GO\,BP\,from\,the\,100\,recruited\,genes\,overlapping\,with\,positive\,set\,pathways/GO\,BP}{Total\,number\,of\,pathways/GO\,BP\,from\,the\,whole\,host\,factors}$$

Descriptive statistics (percentage (%), mean of percentages) and hypothesis testing (student-t test: two-tail assuming unequal variance, one way ANOVA) were derived using R package (Rcmdr)^44^. The deviation from the mean was indicated by standard error bars. P-values < 0.05 at 95% confidence interval were used as a cutoff for the level of significance in Student-t test and one way ANOVA analysis. Similarly, in DAVID KEGG pathway and GO BP analysis, a p-value < 0.05 was used as cutoff value for identification of significantly enriched pathways.

## Electronic supplementary material

supplementary Figures

supplementary Data 1

supplementary Data 2

supplementary Data 3
