# Supplementary material for: Benchmarking selected computational gene network growing tools in context of virus-host interactions
Source: Sci Rep. 2017 Jul 19;7:5805. doi: 10.1038/s41598-017-06020-6 (PMC5517527; doi:10.1038/s41598-017-06020-6)

Supplementary Figures

Benchmarking selected computational gene network growing tools in context of virus-host interactions

Biruhalem Taye, Candida Vaz, Vivek Tanavde, Vladimir A. Kuznetsov, Frank Eisenhaber, Richard J Sugrue, Sebastian Maurer-Stroh

Supplementary Figure S1. Meta-analysis of IHFs from siRNA screening studies. a) number of IHFs shared between the siRNA screening studies b) number of IHFs shared between each siRNA screening study: the higher the number of overlapping genes, the higher the intensity of the color (from 0 overlapping genes = yellow, to 30 = green)

a)

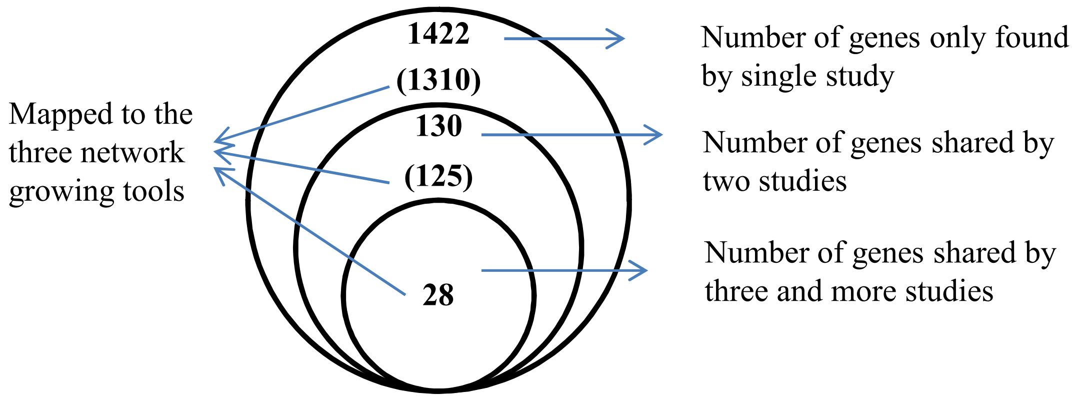

b)

	Atkins et al., 2014	Bakre et al., 2013	Brass et al., 2009	Hao et al., 2008	Karlas et al., 2010	Konig et al., 2010	Shapira et al., 2009	Su et al., 2013	Sui et al., 2009	Tran et a.,l 2013	Watanabe et al., 2014
Atkins et al., 2014		0	0	0	0	0	1	0	0	0	1
Bakre et al., 2013	0		0	0	1	2	2	1	0	1	0
Brass et al., 2009	0	0		12	14	10	11	5	1	2	14
Hao et al., 2008	0	0	12		16	10	2	4	0	2	16
Karlas et al., 2010	0	1	14	16		30	9	7	1	4	21
Konig et al., 2010	0	2	10	10	30		5	7	2	3	14
Shapira et al., 2009	1	2	11	2	9	5		2	2	4	7
Su et al., 2013	0	1	5	4	7	7	2		3	2	1
Sui et al., 2009	0	0	1	0	1	2	2	3		2	2
Tran et al., 2013	0	1	2	2	4	3	4	2	2		0
Watanabe et al., 2014	1	0	14	16	21	14	7	1	2	0	

Supplementary Figure S2. Comparison grown nodes and their edges. a) pair wise analysis of grown genes b) performance comparison using closest possible data sources c) the effect of edges in STRING d) the effect of edges in GeneMANIA e) comparing the mean score of intersecting IHFs in STRING f) comparing the mean score of intersecting IHFs in GeneMANIA

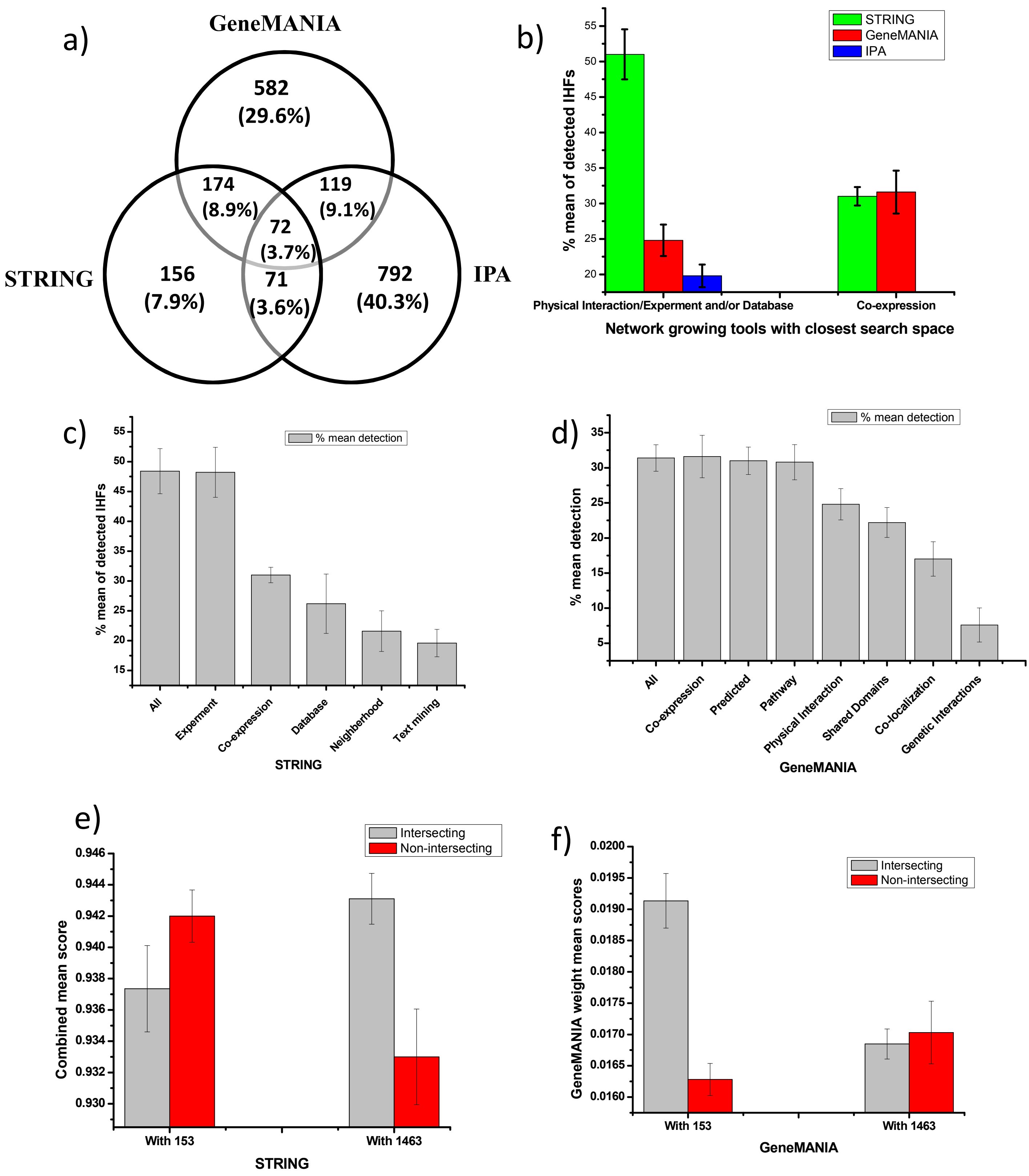

Supplementary Figure S3. **Network topology parameters, GOBP and pathways of grown genes**. a) Average network topology parameters of nodes b) Degree distribution of nodes in GeneMANIA and STRING networks c) Significant pathways of the grown genes overlapping with significant pathways of IHFs d) Significant GOBP of the grown genes overlapping with significant GOBP of IHFs e) performance comparison of STRING new version (STRING v10) and STRING old version (STRING v9).

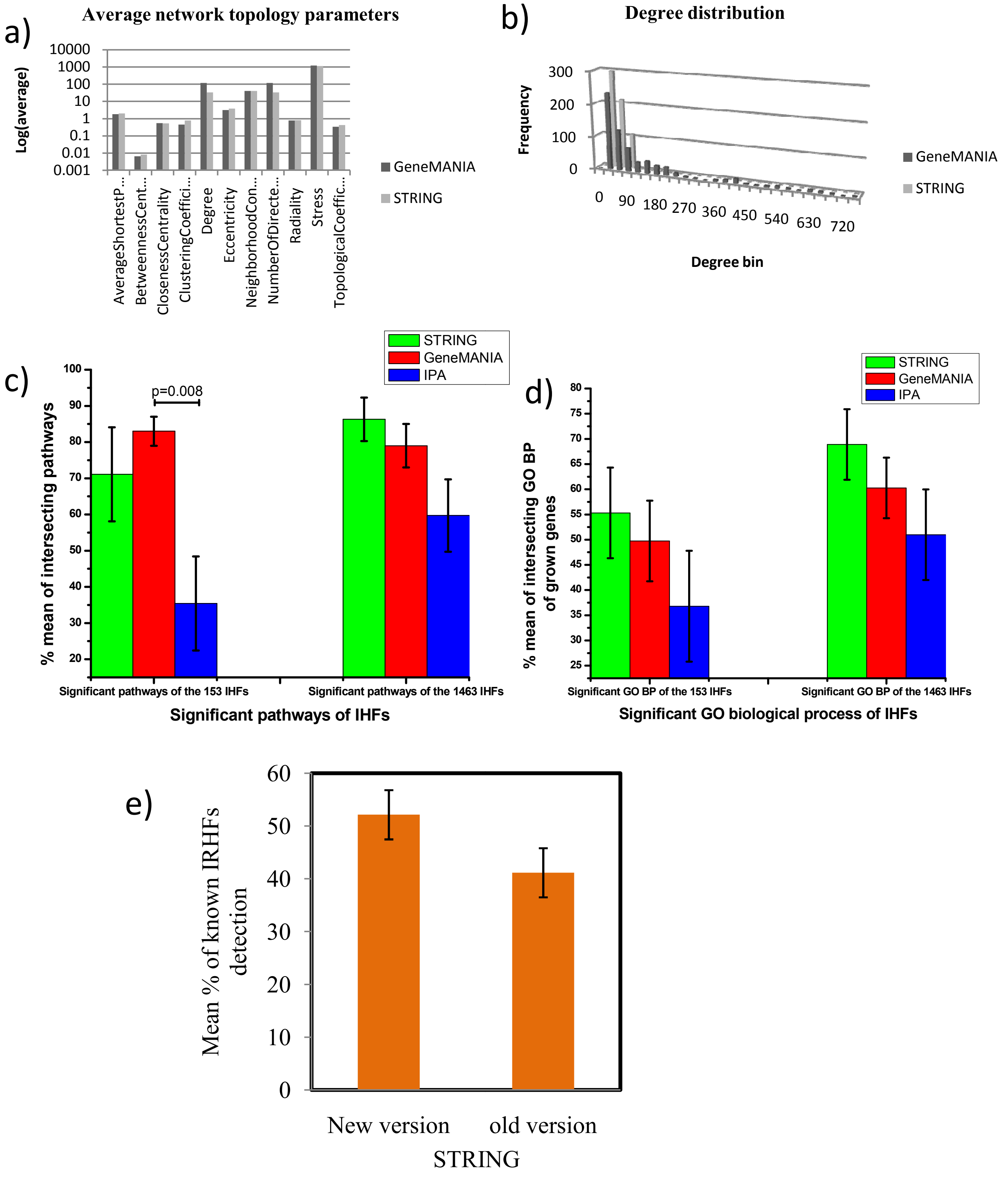

The known and the new candidate host factors could be targeted by FDA-approved drugs

We analyzed 1,445 known IHFs and 1,538 newly identified candidate host targets from network growing (Fig. S4a) using MetaCore^{TM} for therapeutic and secondary drug interactions[1]. All drugs that activate any host factor or have both inhibitory and activation effect were excluded from the analysis. A total of 343 FDA-approved drugs that inhibit 218 host factors were identified (supplementary Data 3). Of these, 288 and 108 drugs targeted 147 known and 71 new host factors, respectively (Fig. S4b; supplementary Data 3). Among the 343 drugs, 53 target both known and new host factors (Fig. S4b). Many drugs (e.g. kinase inhibitors) can target more than one host factor or one host factor can be targeted by many drugs. Overall, 230 host factor targeting FDA approved drugs have been either predicted *in silico* or screened against IAV in previous studies, of which 75 were also predicted by the present study (Fig. S4c, supplementary Data 3). Six of the drugs that have been shown with detailed experimental follow-up to have direct or indirect effect on IAV replication through targeting the IHFs have been also predicted by the current study, suggesting the potential of the approach to identify valid new drugs that could be tested (Fig. S4c, supplementary Data 3).

We have to be aware that network growing provides the functional context of genes being connected but not exactly how they influence each other. For viral infections, these genes could be true host factors (required for the virus) or suppressors of host factors acting as antivirals which would have the opposite effect of helping the virus when targeted. We compiled a list of experimentally identified antiviral genes from the siRNA screens (supplementary Data 1) and found that network growing as well as also experimental screens themselves would identify 2.3% or 1.2% of these antiviral genes with their respective approaches (Fig. S4a). This serves as a

warning that although small in proportions, host networks may include antivirals that should not be targeted. In addition, although the pathways from the new IHFs were indicated to support different stages of IAV replication cycle[2-4], it is worth to validate their effect on IAV.

Among the 204 predicted FDA approved drugs by De Chassey et al., 2012[5] and 60 molecules by Watanabe et al., 2014[2] (in silico against IAV); 71 and eight drugs were also suggested by the MetaCore analysis respectively (supplementary Data 3).While this shows that the approach is in agreement with other studies, in general, it also highlights the diversity of available drug to gene target annotations in different databases (MetaCore in our case, different sources in others). It has been suggested that integration of different databases could improve drug discovery[6].

References

1 MetaCore TM.http://thomsonreuters.com/en/products-services/pharma-life-sciences/pharmaceutical-research/metacore.html. Acess date October 2015.

2 Watanabe, T. *et al.* Influenza virus-host interactome screen as a platform for antiviral drug development. *Cell host & microbe* **16**, 795-805, doi:10.1016/j.chom.2014.11.002 (2014).

3 Karlas, A. *et al.* Genome-wide RNAi screen identifies human host factors crucial for influenza virus replication. *Nature* **463**, 818-822, doi:10.1038/nature08760 (2010).

4 Konig, R. *et al.* Human host factors required for influenza virus replication. *Nature* **463**, 813-817, doi:10.1038/nature08699 (2010).

5 De Chassey, B., Meyniel-Schicklin, L., Aublin-Gex, A., André, P. & Lotteau, V. Genetic screens for the control of influenza virus replication: From meta-analysis to drug discovery. *Molecular BioSystems* **8**, 1297-1303, doi:10.1039/c2mb05416g (2012).

6 Yao, L., Evans, J. A. & Rzhetsky, A. Novel opportunities for computational biology and sociology in drug discovery. *Trends in Biotechnology* **27**, 531-540, doi:10.1016/j.tibtech.2009.06.003 (2009).

Supplementary Figure S4. **The known and the new candidate host factors could be targeted by FDA-approved drugs.** a) Recovery rate of anti-viral host factors in known and new candidate IHFs. b) Number of FDA approved drugs that target the known IHFs, candidate new IHFs and both. c) Number of FDA approved drugs that were shared with previous studies and unique to this study. The red circle shows the number of drugs that have already been experimentally validated to have an effect against influenza.

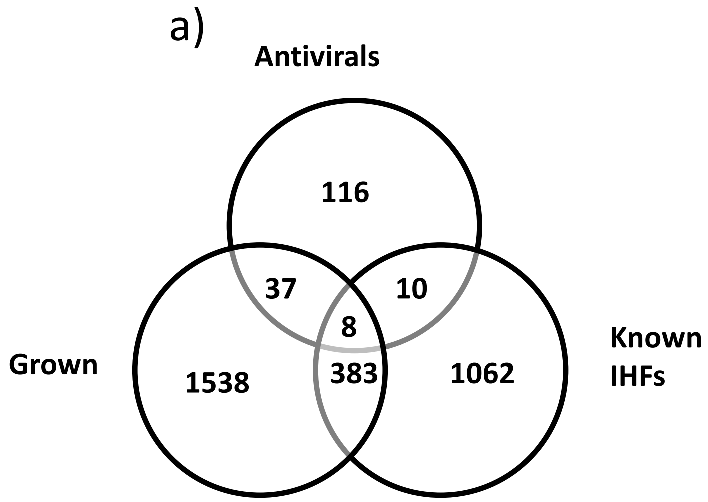

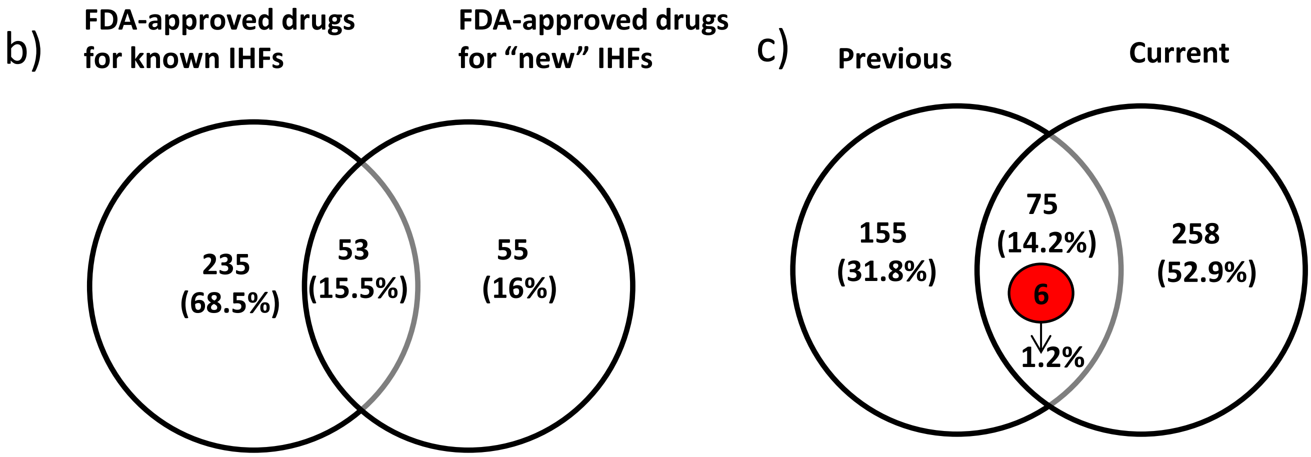

Supplement: Supplementary file 1 — supplementary Figures [file 41598_2017_6020_MOESM1_ESM.pdf]
